# Treatment with OnabotulinumtoxinA for Oromandibular Dystonia: A Systematic Review and Meta-Analysis

**DOI:** 10.3390/toxins16120546

**Published:** 2024-12-16

**Authors:** Kazuya Yoshida, Ryuji Kaji

**Affiliations:** 1 Department of Oral and Maxillofacial Surgery, National Hospital Organization, Kyoto Medical Center, Kyoto 612-8555, Japan; 2 Department of Neurology, Tokushima University, Tokushima 770-8503, Japan; rkaji@tokushima-u.ac.jp

**Keywords:** botulinum toxin therapy, oromandibular dystonia, onabotulinumtoxinA, botulinum toxin, systematic review, meta-analysis, improvement, adverse effect, safety, masticatory muscle

## Abstract

Oromandibular dystonia (OMD) is a focal dystonia characterized by contractions of the masticatory, lingual, and other muscles of the stomatognathic system. We conducted a systematic review and meta-analysis to elucidate the impact and safety of botulinum toxin in OMD. The eligibility criteria were full-length original articles that provided data evaluating the efficacy and adverse effects of onabotulinumtoxinA injections in patients with OMD. PubMed and Embase were searched for articles published before 31 May 2023. We analyzed cases that showed a favorable response (>0% improvement), moderate or greater response (>50% improvement), and adverse effects. A fixed-model meta-analysis of 26 studies involving 1103 patients revealed that an overall favorable effect of onabotulinumtoxinA injection was observed in 96.2% (95% confidence interval [CI], 95–97.5%, *p* < 0.00001) of patients, with significant heterogeneity (*p* < 0.00001, I^2^ = 85%). A moderate response (>50% improvement) was observed in 88.9% of patients (95% CI, 87–90.8%, *p* < 0.00001) with significant heterogeneity (*p* < 0.00001, I^2^ = 85%). Adverse effects were detected in 17.8% of patients, and the most common event was dysphagia (10.1%). Our systematic review found that onabotulinumtoxinA injection was effective, with a low rate of side effects. Further randomized controlled trials are required to clarify the evidence-based efficacy and adverse effects.

## 1. Introduction

Dystonia is a hyperkinetic movement disorder characterized by sustained or intermittent muscle contractions, resulting in abnormal repetitive movements and/or postures [1]. Oromandibular dystonia (OMD) is a focal dystonia involving contractions of the masticatory, lingual, and/or other muscles of the stomatognathic system [2,3,4,5]. Symptoms of OMD include masticatory disturbances, biting of the tongue or cheek membrane, limited mouth opening, muscle pain or discomfort, dysphagia, dysarthria, and esthetic problems [2,3,4,5]. These symptoms can significantly affect patients’ daily activities, resulting in social embarrassment, cosmetic disfigurement, and a decline in overall quality of life [2,3,4,5,6,7].

Based on the site and direction of abnormal dystonic movements, OMD is classified into six subtypes: jaw closing, jaw opening, lingual, jaw deviation, jaw protrusion, and lip dystonia [6,7]. However, mixed dystonia, involving two or more subtypes, is also common. The mean age of OMD onset is in the 50 s, while women are approximately twice as likely to be affected as men [4,5,8]. One meta-analysis by Steeves et al. [9] estimated the prevalence of OMD to be 0.52. Due to underdiagnosis or misdiagnosis, previous estimates may have underestimated the prevalence. A recent study [10] reported a much higher crude prevalence of 9.8 per 100,000 persons, with an incidence of 2.0 per 100,000 persons per year.

Botulinum neurotoxin (BoNT) has been used for OMD since the 1980s [11,12,13]. Many studies have documented the use of BoNT therapy for OMD [7,11,12,13,14,15,16,17,18,19,20,21,22,23,24,25,26,27,28,29,30,31,32,33,34,35,36,37,38,39,40,41,42,43,44,45,46,47,48,49,50,51,52,53,54,55,56,57,58,59,60]. Four FDA-approved BoNT formulations are currently commercially available. These formulations include three types of BoNT type A: onabotulinumtoxinA (Botox, AbbVie, Chicago, IL, USA), abobotulinumtoxinA (Dysport, Ipsen-Pharma, London, UK), and incobotulinumtoxinA (Xeomin, Merz Pharma, Frankfurt am Main, Germany). Additionally, rimabotulinumtoxinB (Myobloc in the USA; Supernus Pharmaceuticals, Inc., Rockville, MD, USA; Neurobloc in Europe, Sloan Pharma, Basel, Switzerland) is a BoNT type B preparation [5]. Clinicians have devised various administration techniques that are currently widely used as safe and reliable treatments. However, BoNT therapy for OMD currently lacks official approval in many countries, including those in North America, Europe, and Japan, and further evidence-based data are required to support this approval. In Japan and some other countries, onabotulinumtoxinA has been the most commonly used for OMD, so we thought that the first priority should be to obtain approval for onabotulinumtoxinA.

A previous systematic review by Dadgardoust et al. [3] analyzed 387 cases from nine reports, evaluating both onabotulinumtoxinA and abobotulinumtoxinA for OMD. They concluded that BoNT was effective in reducing dystonic movements and was generally considered safe. However, they also reported a side effect rate of 27.1%. Another systematic review by Comella [2] analyzed 13 studies on BoNT for OMD and stated that it may be the most effective treatment available to improve movement and quality of life in patients but emphasized the need for more controlled trials.

We believe it is necessary to update the data by including recently published studies with large samples and to focus specifically on the use of onabotulinumtoxinA for OMD. Therefore, we conducted a systematic review to evaluate the efficacy and safety of BoNT therapy with onabotulinumtoxinA in OMD.

## 2. Materials and Methods

### 2.1. Methods

The present systematic review and meta-analysis included full-length original articles that provided sufficient data to evaluate the efficacy and adverse effects of BoNT therapy combined with onabotulinumtoxinA in patients with OMD. The included articles had to meet two criteria: (1) evaluated the effects of onabotulinumtoxinA in patients with OMD and (2) used assessment tools or criteria before and after BoNT injection. The protocol for this systematic review was registered and made available to the University Hospital Medical Information Network on 12 August 2023 (registration number: R000059215).

To evaluate the efficacy and safety of BoNT therapy for OMD, we included various study designs, including randomized controlled trials, open-label case series, observational studies, and retrospective chart reviews. However, we excluded abstracts, case reports, case series with fewer than five cases, and studies using abobotulinumtoxinA, incobotulinumtoxin A, and BoNT type B. Duplicate uses of the same data were carefully examined and excluded. In cases in which sufficient information was not provided, we attempted to contact the corresponding author via email. Language restrictions were not imposed.

### 2.2. Search Strategy

This systematic review and meta-analysis followed the 2020 Preferred Reporting Items for Systematic Reviews and Meta-Analyses statement [61] and the Proposed Reporting Checklist for Authors, Editors, and Reviewers of Meta-analyses of Observational Studies [62].

We searched electronic databases (PubMed, Embase, and Google Scholar) for articles published until 31 May 2023. Two authors and a librarian systematically and independently searched for eligible studies. The search keywords included OMD, lingual dystonia, tongue dystonia, lip dystonia, focal dystonia, botulinum toxin type A, botulinum toxin, onabotulinumtoxinA, botulinum toxin therapy, injection, management, treatment, effect, efficacy, safety, improvement, favorable response, complications, side effects, and adverse events. The final PubMed search strategy was as follows: (“oromandibular dystonia” OR “lingual dystonia” OR “tongue dystonia” OR “focal dystonia”) AND (“botulinum toxin type A” OR “botulinum toxin” OR “onabotulinumtoxinA” OR “botulinum toxin”) AND (“botulinum toxin therapy” OR “injection” OR “management” OR “treatment”) AND (“effect” OR “efficacy” OR “safety” OR “improvement” OR “favorable response” OR “complication” OR “side effect” OR “adverse event”). We also manually searched the reference lists of the included articles to identify additional relevant studies.

### 2.3. Outcome Measures

The research teams who studied the patient response in the included manuscripts used specific assessment tools and criteria. A favorable response was defined as cases excluding those in which the treatment was ineffective. Moderate response was defined as cases that showed an improvement of 50% or more (0%, no effect; 100%, complete cure). We converted a Global Rating Scale [12,15,16,19,20] of 3 (moderate improvement in severity and function) or more, a self-rated subjective improvement score [13,16,36,56,57] of 50% or more, a Global Impression Scale [28,37,48,49] score of 2 (moderate improvement) or more, and an Oromandibular Dystonia Rating Scale [4] score of 50% or more to a moderate response (Table S1). In several reports [18,22,24,27,42,46], it was not possible to convert this ratio to 50% or more (Table S1). Adverse effects were evaluated in all patients in the selected articles. To assess methodological quality, we used the Newcastle–Ottawa Scale for cohort studies [63] to assess the included studies. The studies were scored based on their selection, comparability, and exposure. This review did not require institutional review board approval or patient consent due to the nature of this review.

### 2.4. Statistical Method

We used a fixed-effects model with confidence intervals (CIs) for the meta-analysis. Statistical analyses were performed using Review Manager version 5.4 (The Cochrane Collaboration, Oxford, UK). Prior to the analysis, we estimated the standard error using the Agresti–Coullb method, as the most commonly used method (standard error = standard deviation/square root of n) could not be applied to outcomes with a prevalence of 0% [64]. The Z-test was applied to assess statistical significance.

Heterogeneity was evaluated using the I^2^ statistic (range: 0–100%), interpreted as follows: I^2^ = 0% indicated no heterogeneity, 0% < I^2^ < 25% indicated the least heterogeneity, 25% ≤ I^2^ < 50% indicated mild heterogeneity, 50% ≤ I^2^ < 75% indicated moderate heterogeneity, and 75% ≤ I^2^ indicated strong heterogeneity [65]. Publication bias was assessed visually using funnel plots. Begg–Kendall’s test and Eggert’s test were conducted using the “metafor” package on software EZR version 4.2.2 [66].

## 3. Results

### 3.1. Study Selection

**Figure 1:**
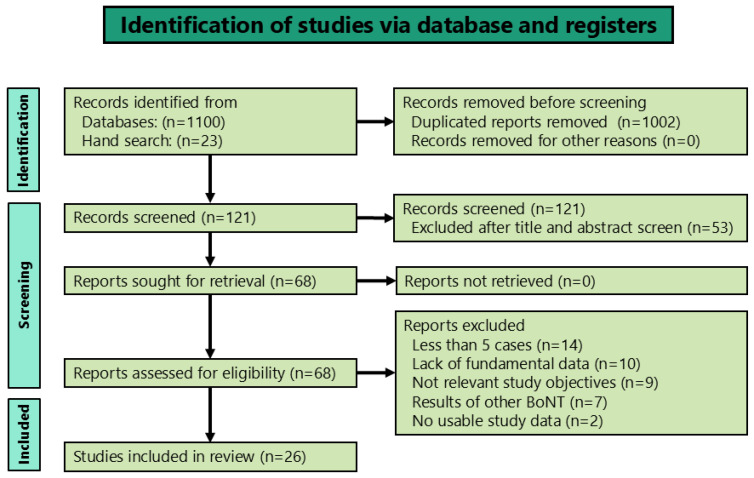
Diagram of the literature search and screening process.

Based on our selection criteria (Figure 1), 26 reports were included, encompassing 1103 patients with OMD treated with BoNT (onabotulinumtoxinA). Only one report was a randomized controlled trial [11], while the remaining 25 were observational studies. Five of these observational studies [12,14,36,47,49] were prospective, whereas the remaining 20 were retrospective. The demographic and clinical data of all studies evaluated in this review are shown in Table S1. Table 1 summarizes the demographics, clinical characteristics, responses, and adverse events associated with the BoNT therapy.

The mean patient age was 54.5 ± 9.1 years. Women comprised the majority (669, or 60.3%) of patients, compared with men (413, or 37.4%). The etiologies of OMD were categorized as idiopathic (58.9%), tardive (24.7%), acquired (12.5%), and unreported (6.5%) (Table 1). The most common subtypes were jaw closing (39.7%), tongue (lingual) (22.8%), jaw opening (17.5%), mixed (6.7%), and jaw deviation dystonia (4.2%) (Table 1). Other associated movement disorders included cervical dystonia (16.4%), blepharospasm (13.9%), limb dystonia (3.9%), spasmodic dysphonia (2.4%), and writer’s cramps (1.4%) (Table 1).

The masseter (48.7%, mean dose: 43.8 units) and lateral pterygoid (25.2%, mean dose: 37.1 units) were the most frequently injected muscles, followed by the genioglossus (23.4%, mean dose: 22.2 units), temporalis (19%, mean dose: 26.2 units), submentalis (16.9%, mean dose: 23.8 units), medial pterygoid (5.4%, mean dose: 19 units), anterior digastric (4.3%, mean dose: 17.1 units), and posterior digastric muscles (2.7%, mean dose: 8.9 units) (Table 1). A total of 196 patients (17.8%) experienced adverse events, most commonly dysphagia (10.1%), dysarthria (0.9%), and pain (0.8%). Less frequent events included chewing difficulties (0.5%) and lip numbness (0.5%). No complications were observed in 881 (79.9%) patients (Table 1).

Various methods have previously been applied to evaluate the effectiveness of BoNTtherapy. These methods can be categorized as rating scales, objective measures, and self-reported improvements. Some examples of rating scales include the Global Rating Scale [11,14,15,18,19], self-rating subjective improvement [12,15,55,56], Global Impression Scale [27,36,47,48], subjective and objective evaluation [17,23], maximal interincisal distance [26], Unified Dystonia Rating Scale [36,47], Glasgow Benefit Inventory [41,54], Burk–Fahn–Marsden Scale [42], Clinical Scoring System [55,56], Sensitive Intelligibility Test [60], and the Oromandibular Dystonia Rating Scale [7] (Table S1).

### 3.2. Meta-Analysis

**Figure 2:**
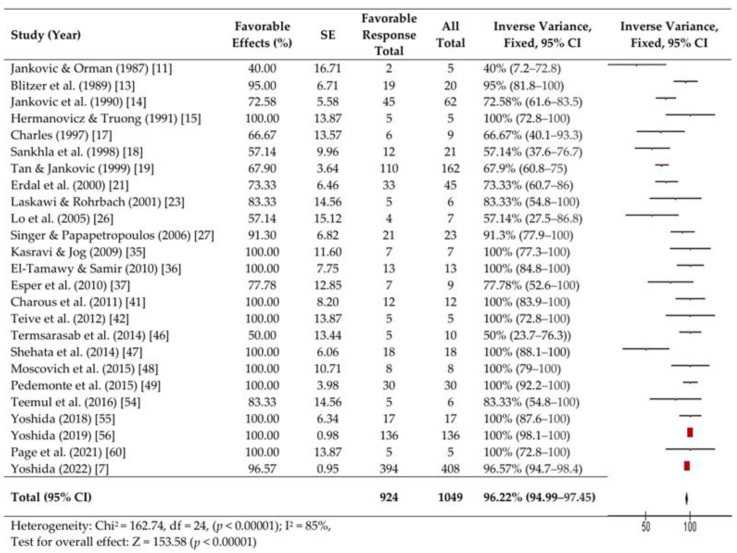
Forest plots of favorable effects. [7,11,13,14,15,17,18,19,21,23,26,27,35,36,37,41,42,46,47,48,49,54,55,56,60].

**Figure 3:**
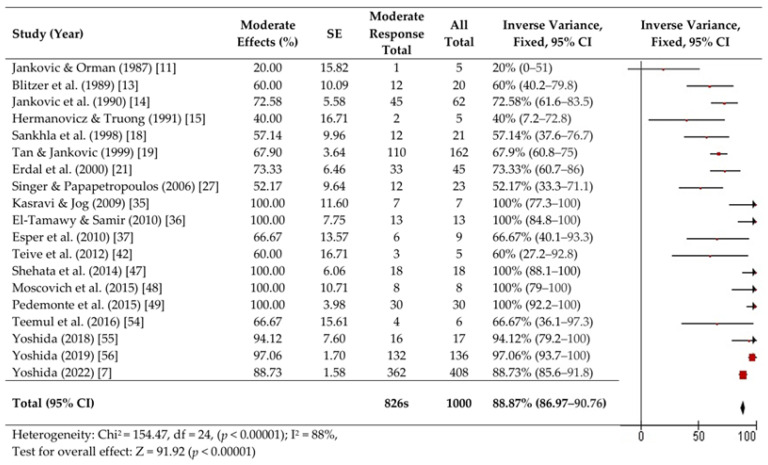
Forest plots of moderate effects. [7,11,13,14,15,18,19,21,27,35,36,37,42,47,48,49,54,55,56].

**Figure 4:**
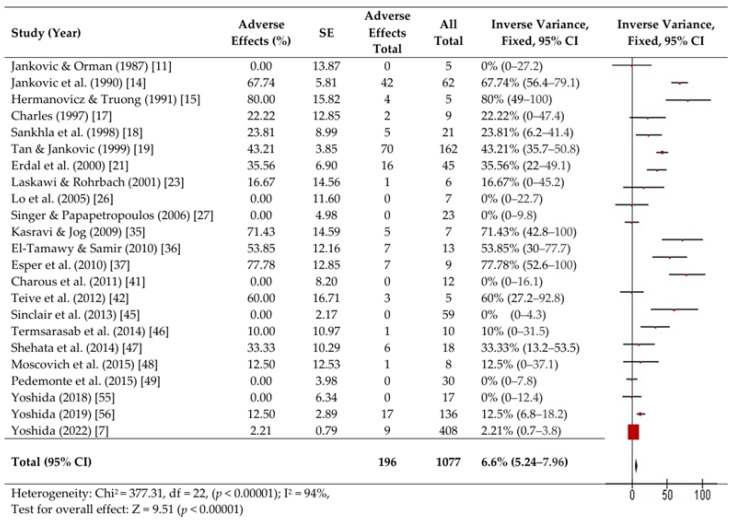
Forest plots of adverse events. [7,11,14,15,17,18,19,21,23,26,27,35,36,37,41,42,45,46,47,48,49,55,56].

We performed a fixed-effects meta-analysis of 26 studies involving 1103 patients with OMD. Forest plots are presented in Figure 2, Figure 3 and Figure 4.

A high proportion of patients (924, 96.2%) achieved a favorable response (>0% improvement) (95% CI, 95–97.5%). However, significant heterogeneity was observed (Chi^2^ = 162.74, df = 24 [*p* < 0.00001]; I^2^ = 85%). The Z-test confirmed statistically significant improvement (Z = 153.58, *p* < 0.00001) (Figure 2). Moderate responses (>50% improvement) were evaluated in 19 reports and were obtained in 826 patients (88.9%, 95% CI, 87–90.8%; Heterogeneity: Chi^2^ = 154.47, df = 18 [*p* < 0.00001]; I^2^ = 88%, Z = 91.92) *p* < 0.00001)) (Figure 3).

Adverse events were examined in 23 reports and detected in 196 patients (6.6%, 95% CI, 5.2–8%; Heterogeneity: Chi^2^ = 373.31, df = 22 [*p* < 0.00001]; I^2^ = 94%, Z = 9.51 (*p* < 0.00001) (Figure 4).

### 3.3. Publication Bias

**Figure 5:**
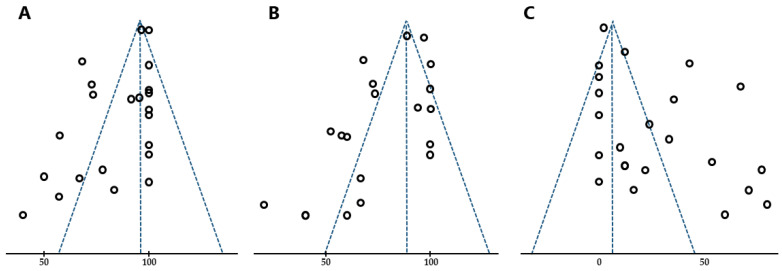
Funnel plots of favorable (**A**) and moderate (**B**) effects and adverse events (**C**).

Funnel plots for the meta-analysis indicated potential publication bias, with an asymmetric distribution. Begg–Kendall’s and Egger’s tests provided some evidence of publication bias for the analyses, described below as follows: favorable response (Begg–Kendall’s test, τ = −0.2770, *p* = 0.0549; Egger’s test, z = −1.8834, *p* = 0.0596) (Figure 5A), moderate response (Begg–Kendall’s test, τ = −0.2111, *p* = 0.2076, Egger’s test, z = −2.9247, *p* = 0.0034) (Figure 5B), and adverse events (Begg–Kendall’s test, τ = 0.2603, *p* = 0.0303, Egger’s test, z = 2.3391, *p* = 0.0193) (Figure 5C).

## 4. Discussion

### 4.1. Results of the Present Study

This systematic review is the first to analyze the response to, and adverse effects of, onabotulinumtoxinA in OMD. Our findings demonstrated significant efficacy: 96.2% of patients experienced a favorable response (>0% improvement), while 88.9% achieved moderate improvement (>50% improvement). As various evaluation methods were used in each report, it was impossible to use a fixed conversion method. In order to analyze the results of as many cases as possible, we analyzed the equivalent 50% for most evaluation methods. Additionally, the rate of adverse effects (17.8%) indicated a favorable safety profile. These results support the use of onabotulinumtoxinA as an effective and safe treatment option for OMD.

A prior systematic review [3] encompassing both onabotulinumtoxinA and abobotulinumtoxinA reported a 49.8% favorable response rate across nine studies with 387 patients. Comella [2] analyzed 13 studies and concluded that BoNT injections may be the most effective treatment available but emphasized the need for more controlled trials. Our study, focusing solely on onabotulinumtoxinA and including 1103 patients, observed a much higher favorable response rate (96.2%).

A previous systematic review also reported a side effect rate of 27.1%, with dysphagia being the most common [3]. Other previously reported adverse effects of BoNT include temporary regional weakness, tenderness in the injection sites, minor discomfort during chewing, asymmetric smiles, loss of smile, lip numbness, muscle atrophy, paresthesia, difficulty swallowing, mouth dryness, speech changes, nasal speech, headache, hematoma, nasal regurgitation, swelling, bruising, facial asymmetry, transient edema, and pain at the injection site (Table S1). While earlier studies reported severe complications, such as aspiration pneumonia, these are likely attributable to less-developed injection techniques and inadequate dosing. Fortunately, most adverse effects are transient and resolve spontaneously. Furthermore, proper injection techniques are crucial to minimize the risk of complications. Accurate knowledge of the local anatomy of the muscles, nerves, and other tissues is essential to facilitate safe and effective BoNT administration. Prior studies have also shown that precise targeting of the intended muscles with BoNT injections leads to better symptom improvement and a lower risk of complications. Empirical differences in injection techniques may also be associated with the adverse effects [5,7].

### 4.2. Treatment Modalities for OMD

OMD treatment requires a multimodal and highly individualized approach. In addition to BoNT therapy, which is considered first-line treatment, other treatment options include pharmacological interventions, muscle afferent blocks, occlusal splints, and surgical procedures (coronoidotomy) [5]. Deep brain stimulation, which is effective for some intractable movement disorders, has an uncertain efficacy in OMD because the innervation of the oral region is bilateral [67].

Several researchers have previously proposed treatment algorithms and strategies for OMD. Sinclair et al. [45] reported a treatment algorithm, while Bakke et al. [68] and Skármeta et al. [69] both presented clinical strategies for BoNT injections into the oromandibular region. In clinical practice, experienced clinicians individualize treatment for each patient. This involves selection of the target muscles and injection sites and determination of the dose and allocation for each BoNT injection. These decisions are generally based on factors such as patient satisfaction, palpation findings, and EMG measurements. To minimize the risks of adverse effects, cost, and antibody development, the BoNT dose must be limited to the lowest effective dose. Personalized adjustment of target muscles, sites, and doses results in better outcomes than standardized approaches without individualized planning.

### 4.3. Pitfalls of BoNT Therapy for OMD

Despite the widespread use of BoNT therapy for OMD since the 1980s, only one double-blind controlled study has thus far been conducted on this topic [11]. Compared with other focal dystonias, such as cervical dystonia or blepharospasm, there is a notable lack of evidence-based research on OMD [5]. This meta-analysis followed the Proposed Reporting Checklist for Authors, Editors, and Reviewers of Meta-analyses of Observational Studies [62]. The number of samples in each study ranged from 5 to 408, with considerable variation. The disadvantage of the random effects model is that it places relative importance on studies with low weights, making them susceptible to publication bias. Therefore, in this study, we presented the data using a fixed-effects model.

In the future, well-designed randomized controlled trials with larger patient groups and longer follow-up periods will be crucial to determine therapeutic efficacy, optimal dose, duration of effect, adverse effects, brand-specific differences, definite treatment indications, and to establish a standardized BoNT therapy protocol. However, the design of traditional placebo-controlled trials for OMD poses ethical challenges [2]. Patients often travel long distances to see OMD specialists with high expectations, making it difficult to create a control group [5].

Several factors may have contributed to the scarcity of randomized controlled trials on OMD. Traditionally, neurologists treat and study dystonia, focusing on more common forms such as cervical dystonia, blepharospasm, or generalized dystonia. The relative rarity of OMD may have led to less interest from neurologists who may have treated patients with OMD incidentally. Additionally, neurologists may not be familiar with the intricate anatomy and function of the masticatory muscles and related structures within the stomatognathic system. Consequently, few evidence-based studies have been conducted to date. Moreover, while some otorhinolaryngologists [13,23,45] can effectively diagnose dystonic symptoms and inject BoNT into the affected muscles, the region involved in OMD falls within the expertise of oral surgeons or dentists, who have the most in-depth knowledge of its anatomy and function. Therefore, to bridge this gap and advance OMD research, collaboration among neurologists, oral surgeons, dentists, and other medical professionals is essential.

Selecting and injecting the affected masticatory muscles can be challenging. Although the masseter and temporalis are relatively easy to target, particular considerations are generally required. These include functional differences between the superficial and deep layers of the masseter muscle, location of the endplate, and avoidance of the parotid gland [5]. Injection into the lateral pterygoid, medial pterygoid, and tongue muscles is more difficult because of their anatomical locations. Detailed descriptions of the injection techniques for these muscles have been discussed previously [55,56,70].

Recent consensus guidelines [71] have suggested that the lateral pterygoid muscle could be easily approached via an extraoral route without EMG guidance and that differentiating between the lateral and medial pterygoid muscles is unnecessary. An extraoral percutaneous approach through the notch can lead to needle penetration of the parotid gland and subsequent mouth dryness due to the spread of BoNT [72]. Therefore, most OMD specialists favor the intraoral approach [45,55,68,70]. A computer-aided design/computer-assisted manufacturing-derived needle guide can aid in the accurate and safe administration of BoNT to the lateral pterygoid muscle [55]. Sonography is important for identifying target muscles and preventing damage to other tissues. Ultrasonographic guidance is often used to treat cervical dystonia [73,74]. This method is also useful for OMD and is recommended in combination with EMG.

Early studies [11,19] injected BoNTs into the submentalis complex instead of the lateral pterygoid muscles for jaw opening dystonia. Recent studies [55] utilizing individualized injection targeting specific muscles have demonstrated higher success rates and fewer adverse events. These findings indicate that the discrepancies and adverse effects observed in previous studies may be related to the injection technique used. Experienced clinicians should administer appropriate and personalized BoNT doses for each patient.

Another limitation of this study is the scarcity of rating scales designed specifically for OMD. The significant variation in symptoms across OMD subtypes makes comprehensive assessment of disease severity and treatment responses challenging. Merz et al. [38] developed and validated an Oromandibular Dystonia Questionnaire in 2010. More recently, the Oromandibular Dystonia Rating Scale was established as a validated tool for OMD assessment [6]. This scale provides a comprehensive evaluation of disease severity, disability, psychosocial functioning, quality of life, and treatment response. A study comparing Oromandibular Dystonia Rating Scale scores before and after BoNT therapy in 408 patients demonstrated a significant improvement (149.1 vs. 57.6) [7]. Similarly, all Oromandibular Dystonia Rating Scale subscales, including examiner-rated (severity, disability, and pain) and patient-rated parameters (general, eating, speech, cosmetic, social/family life, sleep, annoyance, mood, and psychosocial functioning) revealed significant improvements from baseline to four weeks after BoNT therapy. Clinicians should utilize validated OMD-specific rating scales for the objective evaluation of disease severity, disability, psychosocial functioning, and the impact on quality of life.

## 5. Conclusions

This systematic review and meta-analysis indicate that BoNT therapy is a safe and effective treatment for most patients with OMD, inflicting minimal side effects. However, further well-designed trials are necessary to achieve high evidence levels and facilitate formal approval of BoNT for this specific application.

## Figures and Tables

**Table 1:** Demographics, clinical characteristics, and results of BoNT therapy.

Age (years) [mean ± SD]	54.5 ± 9.1	
Sex, (*n* [%])	Women Men NR	669 (60.3) 413 (37.4) 21 (1.9)
Etiology, (*n* [%])	Idiopathic Tardive Acquired NR	650 (58.9) 272 (24.7) 138 (12.5) 72 (6.5)
Subtype of OMD, (*n* [%])	Jaw closing dystonia Tongue dystonia Jaw opening dystonia Mixed dystonia Jaw deviation dystonia Jaw protrusion dystonia Lip dystonia Perioral dystonia NR	438 (39.7) 252 (22.8) 193 (17.5) 74 (6.7) 46 (4.2) 18 (1.6) 13 (1.2) 1 (0.1) 88 (8)
Other dystonia, (*n* [%])	Cervical dystonia Blepharospasm Limb dystonia Spasmodic dysphonia Writer‘s cramp Generalized dystonia Embouchure dystonia Other NR	181 (16.4) 153 (13.9) 43 (3.9) 27 (2.4) 15 (1.4) 10 (0.9) 3 (0.3) 2 (0.2) 208 (18.9)
Number of injections (times) [mean ± SD]	4.1 ± 2.8	
Muscles injected, (*n* [%])	Masseter Lateral pterygoid Genioglossus Temporalis Submentalis Medial pterygoid Anterior digastric Posterior digastric Orbicularis oris Risorius Mentalis Platysma Sternocleidomastoid Other NR	537 (48.7) 278 (25.2) 258 (23.4) 210 (19) 186 (16.9) 60 (5.4) 47 (4.3) 30 (2.7) 29 (2.6) 17 (1.5) 15 (1.4) 14 (1.3) 12 (1.1) 46 (4.2) 31 (2.8)
Dose (units) [mean ± SD]	Masseter Lateral pterygoid Genioglossus Temporalis Submentalis Medial pterygoid Anterior digastric Posterior digastric Platysma	43.8 (35.7) 37.1 (48.9) 22.2 (12.6) 26.2 (14.3) 23.8 (9.2) 19 (5.7) 17.1 (7.9) 8.9 (4.6) 12.3 (6.7)
Adverse events, (*n* [%])	Dysphagia Dysarthria Pain Chewing difficulty Lip numbness Swelling None Other	111 (10.1) 10 (0.9) 9 (0.8) 5 (0.5) 5 (0.5) 2 (0.2) 881 (79.9) 37 (3.4)

OMD, oromandibular dystonia; NR, not reported.

## Data Availability

The original contributions presented in this study are included in the article/Supplementary Materials; further inquiries can be directed to the corresponding author.

## Supplementary Materials

The following supporting information can be downloaded from https://www.mdpi.com/article/10.3390/toxins16120546/s1, Table S1: Summary of demographics, clinical characteristics, and BoNT results in all studies.
