# Supplementary material for: Treatment with OnabotulinumtoxinA for Oromandibular Dystonia: A Systematic Review and Meta-Analysis
Source: Toxins (Basel). 2024 Dec 16;16(12):546. doi: 10.3390/toxins16120546 (PMC11679302; doi:10.3390/toxins16120546)
Supplement: Supplementary file 1 [file toxins-16-00546-s001.zip › toxins-3287061-supplementary.pdf]

Supplemental table S1. Summary of demographics, clinical characteristics, and results of BoNT in all studies

Authors (year of publication)	Patient, n	Design	Age (SD)	Sex	Etiology, n (%)	Subtype, n (%)	Other dystonia, n (%)	Muscle injected, n (%)	Mean number of injections, n (SD)	Average dose, n (SD)	Method for evaluating effect	Therapeutic effect	Favorable response (%)	Moderate response (%)	Subjective improvement (0-100% [SD])	Change from the baseline, % (SD)	Complication
Jankovic & Orman (1987)[11]	5	randomized controlled trial	50.8 (14.9)	W, 3; M, 2	idiopathic, 4 (80); tardive, 1 (20)	NR	Meige, 4 (80); CD, 3 (60); SpD, 1 (20)	M, 3 (60); Scm, 3 (60), Tra, 1 (20)	1.2 (0.45)	M, 45.8 (7.2); Scm, 50; Tra, 50	Global Rating Scale (0 = no effect; 1 = mild effect, but no improvement in function; 2 = moderate improvement, but no change in functional disability; 3 = moderate improvement in severity and function; and 4 = marked improvement in severity and function)	4, 1 (20); 2, 1 (20); 0, 3 (60)	40	20	NR	24.3 (22)	none
Blitzer et al. (1989)[13]	20	prospective	59.7 (12.9)	W, 17; M, 3	idiopathic, 16 (80); acquired, 3 (15); tardive, 1 (5);	JCD, 13 (65); MD, 6,(30); JOD, 1 (5)	Meige, 11 (55); CD, 5 25); generalized, 3 (15)	M, 18 (90), T, 12 (60); Lpt, 6 (30), Oo, 4 (20); Mpt, 2 (10), Gg, 2 (10); Plat, 2 (10); other, 2 (10)	NR	10-40 units per muscle	self-rating subjective improvement (0-100%)	75% or more, 9 (45); 50%, 3 (15); 10-20%, 1 (5); 0-10%, 6 (30); 0%, 1 (5)	95	60	45 (34.8)	NR	NR
Jankovic et al. (1990)[14]	62	prospective	57.2 (12.1)	W, 44; M, 18	idiopathic, 62 (100)	NR	NR	submentalis, 43 (69.4); M, 42 (67.8); Lpt, 3 (4.8)	6.6	submentalis, 29.2 (12.5); M, 51.9 (23.1); Lpt, 32.9 (11)	Global Rating Scale (0-4)	2.2 (1.5) (Global Rating Scale), Global Rating Scale 2 or higher, 73%	73	73	NR	NR	dysphagia, 23 (37.1); dysarthria, 2 (3.2); difficulty opening mouth, 1 (1.6); drooling, 1 (1.6); dysphonia, 1 (1.6); lip numbness, 1 (1.6); other, 14 (22.6)
Hermanowicz & Truong (1991)[15]	5	retrospective	54.4 (8.1)	W, 4; M, 1	NR	JCD, 4 (80); MD, 1 (20)	NR	M, 4 (80); Mpt, 3 (60); T, 2 (50); Da, 1 (20); Gg, 1 (20)	NR	98 (58.5)	Global Rating Scale (0-4) and self-rating subjective improvement (0-4)	Global Rating Scale; 4, 1 (20); 1, 2, 1 (20); 1, 3 (60) Self-rating subjective improvement; marked, 2 (40); moderate, 1 (20); mild, 2 (40)	100	40	NR	NR	dysphagia, 2 (50); difficulty in chewing, 2 (50); none, 1 (20)
Charles (1997)[17]	9	retrospective	62.3 (9.8)	W, 6; M, 3	NR	TD, 9 (100)	Meige, 6 (66.7)	Gg, 9 (100)	3.9 (5.2)	Gg, 34 (14.2)	the patient's report and the physician's examination	reduction in tongue movements in 6 patients	66.7	NR	NR	NR	mild dysphagia, 2 (22.2)
Sankhla et al. (1998)[18]	21	retrospective	NR	NR	acquired (peripherally induced), 21 (100)	NR	NR	NR	NR	NR	Global Rating Scale (0-4)	functional improvement, 12 (57.1); no functional improvement, 9 (42.9); Global Rating Scale 3 or higher, 57.1%	57.1	57.1	NR	NR	jaw weakness, loss of smile, dysphagia, nasal regurgitation, 5 (23.8)
Tan & Jankovic (1999)[19]	162	retrospective	57.9 (15.3)	W, 111; M, 51	idiopathic, 102 (63); tardive, 37 (22.8); acquired, 23 (14.2)	JCD, 85 (52.5); MD, 39 (24); JOD, 35 (21.6); JDD, 3 (1.9)	CD, 93 (57.4); Ble, 81 (50); limb, 34 (21); SpD, 16 (9.9)	M, 135 (83.3); submentalis (geniohyoid, Da, mylohyoid), 118 (72.8)	M, 6.9; submentalis, 5.6	M, 54.2 (15.2); submentalis, 28.6 (16.7)	Global Rating Scale (0-4)	functional improvement, 110 (67.9); no functional improvement, 52 (32.1); Global Rating Scale 3 or higher, 67.9%	67.9	67.9	NR	NR	dysphagia, 44 (27.2); dysarthria, 7 (4.3); facial swelling, 2 (1.2); difficulty chewing, 2 (1.2); other, 15 (9.3)
Erdal et al. (2000)[21]	45	retrospective	58 (16)	W, 29; M, 16	idiopathic, 21 (46.7); acquired, 17 (37.8); tardive, 7 (15.6)	MD, 13 (28.9); JCD, 11 (24.4); JOD, 7 (15.6); TD, 3 (6.7),	CD, 14 (31.1); Ble, 14 (31.1); SpD, 6 (13.3); generalized, 5 (11.1)	Lpt, 124 (total number), thyrohyoid, 105; M, 100; geniohyoid, 100; Da, 99; Mpt, 98; cricopharyngeal, 39; T, 27; Dp, 18; stylohyoid, 13; mylohyoid, 12; sternohyoid, 12;	6.2	Lpt, 16, thyrohyoid, 16; M, 21; geniohyoid, 17; Da, 13; Mpt, 16; cricopharyngeal, 10; T, 20; Dp, 12; stylohyoid, 14; mylohyoid, 16; sternohyoid, 18; omohyoid, 15	NR	at least one effective treatment in 33 patients	73.3	NR	NR	NR	16 patients (35.6), dysphasia, 9% of injection; dysarthria, 2.2%; hematoma, 1.1%; other, 2.2%

								omohyoid, 5; other, 12									
Laskawi & Rohrbach (2001) [23]	6	retrospective	61.5 (14.9)	W, 3; M, 3	NR	JCD, 2 (33.3); JOD, 2 (33.3); TD, 1 (16.7); MD, 1 (16.7)	CD, 1 (16.7)	mouth floor muscles (mylohyoid, geniohyoid, and digastric muscles), 4 (66.7); Gg, 4 (66.7); M, 2 (33.3); Plat, 2 (33.3); Lpt, 1 (16.7); Oo, 1 (16.7)	NR	mouth floor muscles, 10.6 (5.8); Gg, 15 (5.8); M, 4.2 (1.4); Plat, 17; Lpt, 7.5; Oo, 24.2	subjective and objective (inspection, palpation, audio examination) evaluation	subjective improvement in five of six patients	83.3	NR	NR	NR	mild dysphagia, 1 (16.7)
Lo et al. (2005)[26]	7	retrospective	44.8 (15)	W, 4; M, 3	acquired (acute brain injury), 7 (100)	JCD, 7 (100)	CD, 2 (28.6); Ble, 1 (14.3)	M, 7 (100)	1	M, 50	maximal interincisal distance 2.5cm or more	maximal interincisal distance 2.5cm or more in four of seven patients	42.9	NR	NR	NR	none
Singer & Papapetropoulos (2006)[27]	23	retrospective	56 (13.5)	W, 12; M, 11	idiopathic, 23 (100)	JOD, 12 (52.2); JCD, 11 (47.8)	CD, 7 (30.4); facial, 1 (4.3); laryngeal, 1 (4.3); limb, 1 (4.3)	Lpt, 12 (52.2); M, 11 (47.8)	NR	Lpt, 196.4 (141.7); M, 150 (94.3)	Global Impression Scale (0-3) (0 = no improvement; 1 = mild improvement; 2 = moderate improvement; 3 = marked improvement)	marked, 3 (13); moderate, 9 (39.1); mild, 3 (13); no, 2 (8.7)	91.3	52.2	NR	NR	none
Kasravi & Jog (2009)[35]	7	retrospective	67.3 (13.7)	W, 5; M, 2	acquired, 4 (57.1); idiopathic, 2 (28.6); tardive, 1 (14.3)	TD, 7 (100)	NR	Gg, 7 (100)	1-20 times	Gg, 12.5 (6.3)	subjective 5 point scale	5, 2 (28.6); 4, 3 (42.9); 3.5, 1 (14.3); 3, 1 (14.3)	100	100	NR	NR	mild dysphasia, 4 (57.1); moderate dysphasia, 1 (14.3); none 2 (28.6)
El-Tamawy & Samir (2010)[36]	13	prospective	41.5 (6.3)	W, 5; M, 8	acquired, 8 (61.5); idiopathic, 4 (30.8), tardive, 1 (7.7)	JCD, 8 (61.5); JOD, 4 (30.8); MD, 1 (7.7)	CD, 1 (7.7)	M, 8 (61.5); T, 8 (61.5); submentalis, 5 (38.5); Da, 5 (38.5); Lpt, 2 (15.4); Mpt, 2 (15.4)	1	M, 61.3 (11.6); T, 46.9 (8.8); submentalis, 29 (2.2); Da, 25 (3.5); Lpt, 27.5 (10.6); Mpt, 25 (7.1)	Global Impression Scale (0-3) and Unified Dystonia Rating Scale	global impression scale; 3, 8 (61.5); 2, 5 (38.5) Unified Dystonia Rating Scale; 0, 5 (38.5); 1.5, 4 (30.8); 2.0, 2 (15.4); 2.5, 2 (15.4)	100	100	NR	NR	pain at site of injection, 5 (38.5); lip numbness, 2 (15.4)
Esper et al. (2010) [37]	9	retrospective	57.3 (20.3)	W, 5; M, 4	idiopathic, 4 (44.4); tardive, 3 (33.3); acquired, 2 (22.2)	TD, 9 (100)	CD, 1 (11.1); generalized, 1 (11.1)	Gg, 9 (100)	9.9 (17.4)	Gg, 13.3 (8.8)	questionnaire and physician interview (excellent, moderate, mild, and none)	excellent, 5 (55.6); moderate, 1 (11.1); mild, 1 (11.1); none, 2 (22.2)	77.8	66.7	NR	NR	dysphagia, 4 (44.4); mild-to-severe dysarthria, 1 (11.1); facial bruise, 1 (11.1); mild dry mouth, 1 (11.1)
Charous et al. (2011)[41]	12	retrospective	60.6 (10)	W, 8; M, 4	NR	JOD, 12 (100)	NR	Lpt, 12 (100), submentalis, ?	NR	Lpt, 40; submentalis, 10	Modified Glasgow Benefit Inventory (general, support, and physical scores)	37.7 (16.5) (Glasgow Benefit Inventory)	100	NR	NR	NR	none
Teive et al. (2012) [42]	5	retrospective	29.2 (5.3)	W, 2; M, 3	acquired (Wilson's disease), 5 (100)	JOD, 5 (100)	NR	Lpt, 5 (100); submentalis, 5 (100)	NR	Lpt, 35; submentalis, 30	sub-item "mouth" of the Burke-Fahn-Marsden Scale (0-8)	partial reduction of score in all patients	100	60	NR	NR	mild dysphagia, 3 (60)
Sinclair et al. (2013)[45]	59	retrospective	56.6 (14)	W, 40; M, 19	idiopathic, 54 (91.5); acquired or tardive, 5 (8.5)	JCD, 28 (47.5); JOD, 21 (35.6); JDD, 10 (16.9)	two thirds, segmental or generalized dystonia	Lpt, 49 (83.1), M, 31 (52.5); T, 25 (42.4); Da, 17, (28.8); Plat, 7 (11.9); Mpt, 2 (3.4)	5	Lpt, 9.6 (4.3), M, 19.7 (7.2); T, 15.8 (7.1); Da, 4.6, (1); Plat, 7.5 (2.2); Mpt, 20 (0)	NR	NR	NR	NR	NR	NR	none
Termsarasab et al. (2014)[46]	10	retrospective	49.4 (7.7)	W, 7; M, 3	idiopathic, 10 (100)	MD, 4 (40); JOD, 2 (20); JPD, 2 (20); POD, 1 (10)	NR	mostly Lpt or Mpt	NR	NR	NR	mild response, 3 (30); no response, 5 (50); unknown, 2 (20)	50	0	NR	NR	marked dysphagia, 1 (10)
Shehata et al. (2014)[47]	18	prospective	41.9 (6.7)	W, 12; M, 6	acquired (chronic khat chewer), 18 (100)	JOD, 9 (50); JCD, 7 (38.9); MD, 2 (11.1)	CD, 2 (11.1)	Da, 11(61.1); submentalis, 11 (61.1); M, 7 (38.9); T, 7 (38.9); Lpt, 4 (22.2); Mpt, 2(11.1)	1	Da, 25 (3.5); submentalis, 29 (2.2); M, 61.3 (11.6); T, 46.9 (8.8); Lpt, 27.5 (10.6); Mpt, 25 (7.1)	Global Impression Scale (0-3) and Unified Dystonia Rating Scale	marked improvement, 10 (55.6); moderate improvement, 8 (44.4)(Global Impression Scale)	100	100	NR	NR	pain at site of injection, 4 (22.2); lip numbness, 2 (11.1)
Moscovich et al. (2015)[48]	8	retrospective	67 (10.2)	W, 6; M, 2	NR	JDD, 5 (62.5); JDD + JOD, 2 (25), JPD, 1 (12.5)	NR	Lpt, 8 (100)	NR	NR	Clinical Global Impression Scale	very much improved, 6 (75); much improved, 2 (25) (Clinical Global Impression Scale)	100	100	NR	NR	nasal speech, 1 (12.5)
Pedemonte et al. (2015)[49]	30	prospective	NR	M, 30	acquired (post-traumatic), 30	JCD, 30 (100)	NR	M, 30 (100), T, 30 (100)	2 (0.72)	M, 30; T, 20	questionnaire (0-3) (bruxism [0 or 1], involuntary muscle	decrease of bruxism, involuntary muscle	100	100	NR	NR	None

					(100)						contraction [0 or 1], and muscle pain [0 or 1])	contraction, and muscle pain in all patients					
Teemul et al. (2016)[54]	6	retrospective	61 (17.6)	W, 5	NR	MD, 3 (50), JDD, 2 (33.3); JPD, 1 (16.7)	Ble, 1 (16.7)	Lpt, 5 (83.3); M, 3 (50); T, 3 (50); Da, 1 (16.7); Gg, 1 (16.7)	NR	Lpt, 24 (2.2); M, 20 (5); T, 15 (8.7); Da, 15; Gg, 20	Glasgow Benefit Inventory	a good outcome in five of six patients	83.3	66.7	NR	82.2 (15.5)	NR
Yoshida (2018)[55]	17	retrospective	48.5 (12.8)	W, 6; M, 11	idiopathic, 10 (58.8); tardive, 7 (41.2)	JOD, 17 (100)	CD, 4 (23.5); WC, 3 (17.6); Ble, 2 (11.8); generalized, 1 (5.9)	Lpt, 17 (100)	2.5 (1.6)	Lpt, 28 (5.8)	self-rating scale (0-100) and Clinical Scoring System (0-16) (mastication [0-4], speech [0-4], pain [0-4], and discomfort [0-4])	66.3 (16.2) percent of improvement by Clinical Scoring System (mastication, speech, pain, and discomfort)	100	93.8	66.9 (14.4)	66.3 (16.2)	None
Yoshida (2019)[56]	136	retrospective	46.5 (13.5)	W, 74; M, 62	idiopathic, 94 (69.2); tardive, 42 (30.8)	TD, 136 (100) (JOD, 15 (11); JCD, 8 (5.9); JDD, 3 (2.2), JPD, 1 (0.7)	Ble, 7 (5.1); WC, 5 (3.4); CD, 5 (3.4); EmD, 1 (0.7); SpD, 1 (0.7)	extrinsic and intrinsic muscles, 136 (100); Gg, 123 (93.2); Lpt, 24; M, 23; Dp, 8; Mpt, 5; T, 4; other, 17	4.8 (3.9)	extrinsic and intrinsic muscles (bilateral), 43.1 (5.3); Gg (bilateral), 45.4 (4.2); Lpt, 22.3 (4.1) ; M, 25.1 (3.6); Dp, 5.5 (2.1); Mpt, 10.2 (3.6); T, 18.5 (5.2)	self-rating scale (0-100) and Clinical Scoring System (mastication, speech, pain, and discomfort)	77.6 (16.7) percent of improvement by Clinical Scoring System	100	97.1	79.0 (16.0)	77.6 (16.7)	mild and transient dysphagia, 17 (12.5)
Page et al. (2021)[60]	5	retrospective	65.6 (14.2)	W, 2; M, 3	NR	MD, 2 (40); JCD, 1 (20); TD, 1 (20); JOD, 1 (20)	Meige, 1 (20)	M, 3 (60); Mpt, 2 (40); Da, 2 (40); Lpt, 1 (20): Gg, 1 (20); Oo, 1 (20)	NR	M, 18.3 (2.9); Mpt, 17.5 (3.5); Da, 20; Lpt, 15: Gg, 15; Oo, 60	Sentence Intelligibility Test	some degree of benefit in all patients	100	100	NR	NR	NR
Yoshida (2022)[7]	408	retrospective	52 (15.6)	W, 262; M, 146	idiopathic, 248 (58.8); tardive, 168 (41.2)	JCD, 223 (54.7); TD, 86 (21.1); JOD, 50 (12.3); JDD, 23 (5.6); JPD,13 (3.2); LD, 13 (3.2)	CD, 43 (10.5); Ble, 25 (6.1); limb, 8 (2); WC, 7 (1.7); SpD, 3 (0.7), EmD, 2 (0.5)	M, 210 (51.5); Lpt, 129 (31.6); T, 119 (29.2); Gg, 101 (24.8); Mpt, 42 (10.3); Oo, 23 (5.7); Dp, 22 (5.4); Ris, 17 (4.2); Ment, 15 (3.7); Da, 10 (2.5); Scm, 9 (2.2); Zm, 3 (0.7); Plat, 3 (0.7); others, 23 (5.6)	5.4 (5)	NR	Oromandibular Dystonia Rating Scale (0-314)	96.6%, responders (≥30% improvement in the total OMDRS score) 3.6%, partial responders (<30% improvement)	96.6	88	NR	63.1 (18.6)	mild or transient difficulty in swallowing, 9 (2.1)

W, woman; M, man; NR, not reported; JCD, jaw closing dystonia; MD, mixed dystonia, JOD, jaw opening dystonia; TD, tongue dystonia, JDD, jaw deviation dystonia; JPD, jaw protrusion dystonia; LD, lip dystonia; POD, perioral dystonia, CD, cervical dystonia; Ble blepharospasm; SpD, spasmodic dysphonia; EmD, embouchure dystonia; M, masseter; T, temporalis; Scm, sternocleidomastoid; Tra, trapezius; Lpt, lateral pterygoid; Mpt, medial pterygoid; Oo, orbicularis oris; Gg, genioglossal; Plat, platysma; Da, anterior digastric; Dp, posterior digastric; Ment, mentalis; Ris, risorius; Zm, zygomatic major.
